# A novel method, digital genome scanning detects *KRAS *gene amplification in gastric cancers: involvement of overexpressed wild-type KRAS in downstream signaling and cancer cell growth

**DOI:** 10.1186/1471-2407-9-198

**Published:** 2009-06-23

**Authors:** Hiroaki Mita, Minoru Toyota, Fumio Aoki, Hirofumi Akashi, Reo Maruyama, Yasushi Sasaki, Hiromu Suzuki, Masashi Idogawa, Lisa Kashima, Kazuyoshi Yanagihara, Masahiro Fujita, Masao Hosokawa, Masanobu Kusano, Sorin Vasile Sabau, Haruyuki Tatsumi, Kohzoh Imai, Yasuhisa Shinomura, Takashi Tokino

**Affiliations:** 1 Department of Molecular Biology, Cancer Research Institute, Sapporo Medical University, Sapporo, Japan; 2 First Department of Internal Medicine, Sapporo Medical University, Sapporo, Japan; 3 Scholarly Communication Center, Sapporo Medical University, Sapporo, Japan; 4 First Department of Anatomy, Sapporo Medical University, Sapporo, Japan; 5 Department of Ecology and Evolution, State University of New York at Stony Brook, New York, USA; 6 Central Animal Laboratory, National Cancer Center Research Institute, Tokyo, Japan; 7 Keiyukai Sapporo Hospital, Sapporo, Japan; 8 Otaru Kyokai Hospital, Otaru, Japan; 9 Department of Human Science and Informatics, Tokai University, Sapporo, Japan

## Abstract

**Background:**

Gastric cancer is the third most common malignancy affecting the general population worldwide. Aberrant activation of KRAS is a key factor in the development of many types of tumor, however, oncogenic mutations of *KRAS *are infrequent in gastric cancer. We have developed a novel quantitative method of analysis of DNA copy number, termed digital genome scanning (DGS), which is based on the enumeration of short restriction fragments, and does not involve PCR or hybridization. In the current study, we used DGS to survey copy-number alterations in gastric cancer cells.

**Methods:**

DGS of gastric cancer cell lines was performed using the sequences of 5000 to 15000 restriction fragments. We screened 20 gastric cancer cell lines and 86 primary gastric tumors for *KRAS *amplification by quantitative PCR, and investigated *KRAS *amplification at the DNA, mRNA and protein levels by mutational analysis, real-time PCR, immunoblot analysis, GTP-RAS pull-down assay and immunohistochemical analysis. The effect of *KRAS *knock-down on the activation of p44/42 MAP kinase and AKT and on cell growth were examined by immunoblot and colorimetric assay, respectively.

**Results:**

DGS analysis of the HSC45 gastric cancer cell line revealed the amplification of a 500-kb region on chromosome 12p12.1, which contains the *KRAS *gene locus. Amplification of the *KRAS *locus was detected in 15% (3/20) of gastric cancer cell lines (8–18-fold amplification) and 4.7% (4/86) of primary gastric tumors (8–50-fold amplification). *KRAS *mutations were identified in two of the three cell lines in which *KRAS *was amplified, but were not detected in any of the primary tumors. Overexpression of KRAS protein correlated directly with increased *KRAS *copy number. The level of GTP-bound KRAS was elevated following serum stimulation in cells with amplified wild-type *KRAS*, but not in cells with amplified mutant *KRAS*. Knock-down of *KRAS *in gastric cancer cells that carried amplified wild-type *KRAS *resulted in the inhibition of cell growth and suppression of p44/42 MAP kinase and AKT activity.

**Conclusion:**

Our study highlights the utility of DGS for identification of copy-number alterations. Using DGS, we identified *KRAS *as a gene that is amplified in human gastric cancer. We demonstrated that gene amplification likely forms the molecular basis of overactivation of KRAS in gastric cancer. Additional studies using a larger cohort of gastric cancer specimens are required to determine the diagnostic and therapeutic implications of *KRAS *amplification and overexpression.

## Background

Gastric cancer is the third most common malignancy affecting the general population worldwide [1]. Specific genetic changes have been reported in gastric cancer, including the amplifications of *KSAM*, *MET *and *ERBB2*, and mutations in *p53*, *APC*, and *CDH1 *[2]. While gain-of-function mutations of *KRAS *are some of the most commonly observed genetic alterations in a variety of tumors, including pancreatic (60%), biliary tract (33%) and colon (32%) [3], these mutations are infrequent in gastric cancer (2–7%) [4-7]. In general, *RAS *mutations associated with tumorigenesis "lock" RAS in an active GTP-bound state. GTP-RAS binds to a number of effector proteins to stimulate downstream signaling pathways, among which the RAF-MAP kinase cascade and the phosphatidylinositol 3-kinase (PI3K)-AKT pathways of cell growth and oncogenesis are the best characterized [3]. Prolonged activation of RAS can also occur through mechanisms that do not involve mutations in RAS. For example, reduced expression of let-7 microRNAs, which suppresses RAS by targeting the 3'untranslated region of *RAS *mRNAs, is often associated with a higher RAS protein level in tumors [8]. To date, the molecular mechanisms of oncogenic activation of RAS in gastric cancer have not been fully elucidated.

Amplification of genomic sequences containing genes that are critical for cell growth is one of the primary mechanisms of activation of oncogenes in cancer, and is often associated with tumor progression, poor prognosis and/or drug resistance [9]. Of the numerous methods currently available for detecting copy number alterations genome-wide, the current gold standard is the array CGH method (aCGH). Over the past few years, the resolution of aCGH has improved rapidly through the use of oligonucleotide probes, and has surpassed that of aCGH using standard BAC probes [10]. However, aCGH is also susceptible to the inherent noise of hybridization-based intensity measurements, as the signal quality is affected by repetitive sequences and is dependent on probe quality [11]. In fact, optimization of probe design has been a major challenge in the development of tiling arrays [12,13].

Digital karyotyping (DK) was developed by Wang et al. [14], and is not limited by the inherent problems of array techniques. DK involves the digital enumeration of short fragments of genomic DNA (termed tags), providing a quantitative measurement of DNA copy number through tag density analysis along each chromosome. DK has been applied successfully to a variety of tumor types to detect copy-number alterations, including the amplification of *TYMS*, *RSF1 *and *OTX2*, and deletion of *MKK4 *and *dystrophin *[15-19]. Despite the efficiency of DK, it is technically challenging for broad applications, because it involves PCR amplification and the generation of tags of 21-base pairs (bp) in length to precisely represent the chromosome location of interest.

We report here the development of a novel method, termed DGS, for the quantitative analysis of copy number variation, which is based on the tag-counting concept of DK, but uses a simplified process of tag preparation. DGS of gastric cancer cell lines detected the amplification of the *KRAS *locus on chromosome 12p12.1. Our results provide a molecular basis for the overactivation of KRAS, and suggest that the activation of KRAS downstream signaling events may promote gastric cancer cell proliferation.

## Methods

### Cell lines and tissues

The cell lines analyzed in the current study are listed in Additional file 1. The HSC and SH101P4 cell lines were established by Kazuyoshi Yanagihara [20]; all others were obtained from American Type Culture Collection or the Japanese Collection of Research Bioresources (Tokyo, Japan). All cell lines were cultured in the recommended media. For serum stimulation, cells were incubated in media that lacked serum for 24 hours (h), and then either unstimulated, or stimulated for 1 h with media containing 10% fetal calf serum (FCS). Primary gastric cancer specimens were obtained from the Department of Surgery, Keiyukai Sapporo Hospital, with informed consent from each patient. Genomic DNA was extracted using the phenol-chloroform method, followed by RNase treatment. Total RNA was extracted using Trizol (Invitrogen, Carlsbad, CA, USA), according to the manufacturer's instructions. Genomic DNA of normal peripheral blood leukocytes (Biochain, Hayward, CA, USA) and total RNA from normal gastric mucosa from healthy individuals (Biochain and Invitrogen) were purchased. Primary gastric cancers were classified using clinicopathological features, as shown in Additional file 2, according to the pTNM classification scheme (5th edition, 1997) [21] and the Lauren's classification system [22]. *KRAS*-amplification status according to age was compared using the Student t test; according to grade, pT status, pN status, and disease stage using the Mann-Whitney U test; and according to gender, histology and pM status using the Fisher exact test. All tests were 2-tailed, and a *P *value of < 0.05 was considered statistically significant.

### Digital genome scanning

Briefly, 40 μg of genomic DNA were subjected to restriction enzyme digestion using *Mbo*I (Takara, Tokyo, Japan) and then separated by electrophoresis on a 3% Nusieve GTG agarose gel. Short fragments (30–60 bp, termed real tags) were electroeluted, concatenated and subcloned into *Bam*HI-digested pBluescript II KS+ (Stratagene, La Jolla, CA) using Mighty Mix DNA ligation solution (Takara). *Escherichia coli *DH10B were transformed with the recombinant plasmids, the transformants were pooled and the plasmid DNA was purified to generate the 1st library. Concatemers of real tags were excised by *Spe*I/*Pst*I digestion from the 1st library, and fragments in the range of 140 to 800 bp were electroeluted, concatenated and subcloned into pBluescript II KS+ to generate the 2nd library. Second library plasmids containing concatemers of *Spe*I/*Pst*I fragments were sequenced using an ABI3130 Genetic Analyzer (Applied Biosystems, Foster City, CA, USA), according to manufacturer's instructions. Unique real tags were mapped to human chromosome sequences, and tag density, defined as the ratio of real tags to virtual tags over moving windows, was calculated to detect abnormalities in DNA content using threshold values defined by DGS simulations. Tag positions and tag density ratios were visualized using Custom Tracks and Genome Graphs from the University of California, Santa Cruz (UCSC) genome browser (Mar. 2006 freeze, hg18) [23-25]. The detailed protocols for DGS, virtual tag characterization and *in silico *simulations are available in Additional file 3.

### Quantitative real-time PCR

Relative DNA copy number was determined by quantitative real-time PCR using a SYBR Green PCR Master Mix (Applied Biosystems) and the ABI PRISM 7000 (Applied Biosystems). DNA content per haploid genome was normalized to that of a repetitive element, Line-1, and calculated by the comparative CT (ΔΔCT) relative quantification method using the formula 2^(*Nt *- *Nline*)-(*Xt *- *Xline*)^, where *N*_*t *_is the threshold cycle number observed for an experimental primer in normal leukocyte DNA, *N*_*line *_is the threshold cycle number observed for the Line-1 primer in normal leukocyte DNA, *Xt *is the average threshold cycle number observed for the experimental primer in cancer cell DNA, and *X*_*line *_is the average threshold cycle number observed for the Line-1 primer in cancer cell DNA [14]. Genomic amplification was defined as a greater than 4-fold increase in DNA content. The primer sequences for each locus are available in Additional file 4. The allelic proportion of mutant *KRAS *(G12V, ggt→gTt) was determined by employing a modified real-time PCR procedure according to Itabashi *et al *[26]. The detailed protocol is available in Additional file 3. cDNA was prepared using SuperScript III reverse transcriptase (RT, Invitrogen), and the mRNA level of each gene was determined by real-time RT-PCR using the TaqMan Gene Expression Assay (Applied Biosystems). Relative mRNA levels were calculated by the comparative CT method using *GAPDH *as an endogenous control. The primer/probe sets used are shown in Additional file 5.

### Fluorescence *in situ *hybridization (FISH)

BACs that contained the *KRAS *locus (RP11-636P12) and chromosome 12q24.2 (RP11-91M21) were labeled with Cy3 and Cy5, respectively, and then incubated with slides prepared with interphase and metaphase chromosomes. Nuclei were counter-stained with 4',6-diamino-2-phenylindole (DAPI), and slides were analyzed using a fluorescence microscope (Leica CW-4000).

### Mutational analysis of *KRAS *and *PIK3CA*

Amplified genomic fragments were either sequenced directly, or subcloned using the TOPO TA-cloning kit (Invitrogen) and then sequenced. At least ten clones from two independent PCR assays per locus were sequenced using M13 Forward and Reverse primers (Invitrogen). The sequences of the primers used for amplification of *KRAS *(exons 1 and 2) and *PIK3CA *(exons 9 and 20) are shown in Additional file 6.

### Immunoblot analysis

Cells were lysed in Lysis buffer containing 20 mM Tris-HCl (pH7.5) buffer, 150 mM NaCl, 1 mM EDTA, 1% Triton X, 10% glycerol, 10 mM NaF, 1 mM sodium vanadate, 50 mM β-glycerophosphate, 1 mM phenylmethansulfonyl fluoride, 1 mM dithiothreitol, and a protease inhibitor cocktail (Roche, Mannheim, Germany). Proteins were separated by SDS-PAGE and electroblotted onto an Immobilon-P membrane (Millipore, Billerica, MA, USA). The membranes were analyzed by immunoblot using the following antibodies, as indicated: mouse monoclonal anti-KRAS, -NRAS, and -HRAS antibodies (sc-30, sc-31, and sc-29, respectively, Santa Cruz Biotechnology, Santa Cruz, CA, USA); anti-actin antibody (Millipore); rabbit polyclonal anti-p44/42 MAP kinase, -phosho-p44/42 MAP kinase (Thr202/Tyr204), -Akt and -phospho-Akt (Ser473) antisera (Cell Signaling Technology, Danvers, MA, USA).

### GTP-RAS pull-down assay

The activation of RAS was detected using an EZ-Detect Ras Activation Kit (Pierce, Rockford, IL, USA). Briefly, cell lysate (500 μg) was incubated with immobilized Raf1 Ras-binding domain fused to glutathione S-transferase (GST-Raf1-RBD). Precipitates were washed 3 times, and bound proteins were eluted by boiling for 5 minutes (min). Proteins were resolved on a 12% polyacrylamide gel, transferred to an Immobilon-P membrane, and subjected to immunoblot analysis using anti-KRAS, -NRAS, or -HRAS antibodies.

### RNA interference

A custom-designed *KRAS *siRNA (5'-AGAGUGCCUUGACGAUACAdTdT-3'), targeting a region of *KRAS *that is not associated with known oncogenic mutations, was synthesized by Dharmacon (Lafayette, Co, USA). siRNAs targeting *LRMP*, *LYRM5 *and *CASC1 *were purchased from Ambion (No.144181, 284911 and 147715). A universal non-targeting siRNA (non-specific control VII, Dharmacon) was used as a negative control. In each experiment, 5 × 10^6 ^cells were transfected with 7.5 μl of 20 μM siRNA by electroporation (Amaxa, Cologne, Germany) using Nucleofector kit V or T, according to the manufacturer's instructions.

### Cell proliferation assay

Following transfection with siRNAs, the gastric cancer cell lines HSC45, MKN1, AGS and NUGC4 were seeded in 96-well plates at a density of 8000 cells/100 μl in standard medium containing 10% FCS. Cell number at 48, 72 and 96 h post-transfection was determined indirectly by colorimetric assay using Cell Counting Kit-8 solution (Dojindo, Kumamoto, Japan). The assay is based on the reduction of a tetrazolium salt ([2-(2-methoxy-nitrophenyl)-3-(4-nitrophenyl)-5-(2,4-disulfophenyl)-2-tetrazolium, monosodium salt], WST-8) and is used as a measure of live cells. The absorbance of each well at 450 nm was measured using a microplate reader (Model 680, Bio-Rad, Hercules, CA, USA).

### Flow cytometry

Flow cytometry was carried out as described previously [27]. Briefly, adherent and detached cells were harvested, fixed in 90% cold ethanol, treated with RNase A (500 units/ml), and then stained with propidium iodide (50 μg/ml). For each sample, 30000 events were analyzed using the cell cycle analysis platform of FlowJo program (Tree Star, Ashland, OR, USA).

### Immunohistochemistry

Formalin-fixed, paraffin-embedded sections of gastric tumors were deparaffinized, hydrated, and then treated with peroxidase blocking solution (3% H_2_O_2 _in Methanol). Sections were autoclaved at 105°C for 10 min in target retrieval solution (Dako, Glostrup, Denmark). Sections were incubated with a mouse anti-KRAS antibody (1:100 dilution; Santa Cruz Biotechnology) for 1 h at room temperature, and immunoreactivity was detected using ENVISION-Plus reagents (Dako).

## Results

### Digital genome scanning and characterization of virtual tags *in silico*

**Figure 1:**
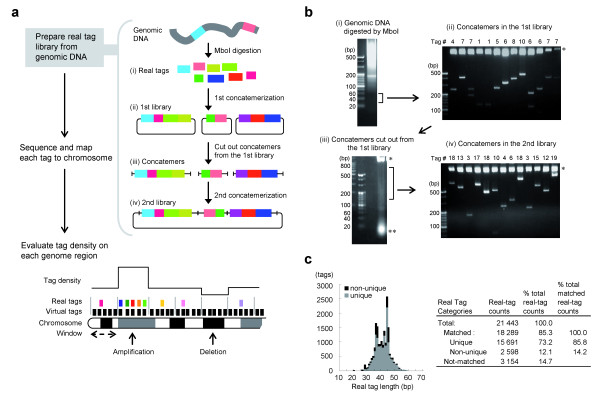
**DGS and preparation of real tags**. (**a**) Schematic outline of DGS. Colored boxes represent genomic *Mbo*I real tags. See text for details. (**b **and **c**) Preparation (**b**) and characterization (**c**) of real tags. Representative results using genomic DNA from MKN1 gastric cancer cells are shown. (**b**) Short fragments of *Mbo*I-digested genomic DNA (30 to 60 bp) were electroeluted from an agarose gel (i), concatenated and subcloned. Resultant recombinant plasmids were pooled to generate the 1st library (ii). Long concatemers (140 to 800 bp) were excised from 1st library vectors, electroeluted (iii), concatenated and subcloned. The resultant recombinant plasmids represent 2nd library clones (iv). The number of tags contained in each clone is shown at the top of each lane. Inserts were examined by *Xho*I/*Sac*I digestion in panels (ii) and (iv). *, vector fragments; **, *Spe*I/*Pst*I digestion of the multiple-cloning site without insert. (**c**) Actual number of real tags from the 2nd library is shown in the histogram (left), and their characteristics are summarized (right).

Digital genome scanning (DGS) is a method of quantitating gene copy number by enumerating short genomic DNA fragments (termed real tags) that are generated experimentally by *Mbo*I endonuclease digestion (Figure 1a). To eliminate the complicated steps involved in tag preparation, we computationally characterized the short DNA fragments that are produced by single restriction enzyme digestion with *Mbo*I, which recognizes the 4-bp sequence GATC. *In silico *digestion of the human genome by *Mbo*I produced approximately 1.6 million restriction fragments (termed virtual tags) in the range of 20–130 bp (Additional file 7a). Nucleotide sequence analysis revealed that approximately 65% of the virtual tags contained repetitive sequences, as defined in the public database of repeat elements (Additional file 7a). Importantly, sequence matching to the human genome database revealed that approximately 85% of the virtual tags mapped uniquely to precise chromosomal locations (Additional file 7b, c). Even if the virtual tags include repetitive sequences in part, approximately 80% of the repetitive tags turned out to be unique. The average distance between two unique virtual tags of 30 to 60 bp in length was 7.6 kb, the median distance was 4.5 kb and 97.8% of intervals were shorter than 30 kb (Additional file 7d). Similar tag interval characteristics were observed for virtual tags the range of 70 to 100 bp (average distance, 7.9 kb; median distance, 4.8 kb; 97.4% were shorter than 30 kb), and 100 to 130 bp (average distance, 7.9 kb; median distance, 4.9 kb; 97.4% were shorter than 30 kb (Additional file 7e, f). Furthermore, the density of unique virtual tags was nearly equal in each chromosome (Additional file 7g). These *in silico *findings suggested that the majority of short *Mbo*I tags would be informative for DGS.

### DGS simulation *in silico*

The ability of DGS to detect genome-wide changes is based on genome characteristics, such as the copy number and the size of the alteration, and the number of real tags obtained from sequence analysis. To predict the size of alteration that could reliably be detected, given a fixed number of computationally sampled tags, we used Monte Carlo simulation to calculate a positive predictive value (PPV), which is the probability that a detected alteration represents a true alteration. For example, we found that an analysis of 5000 tags could reliably detect a 10-fold amplification of 500 kb, a homozygous deletion of 7.5 Mb, or a single copy loss of a 30 Mb region, but could not detect a subchromosomal gain smaller than 30 Mb (Additional file 8). Both the sensitivity and specificity of detecting these types of alteration were >99% in cases with high PPVs (>90%), which indicated that neither was a limiting factor in this analysis (data not shown).

### Preparation of real tags from human genomic DNA

For DGS of the gastric cancer cell lines HSC45 and MKN1, we prepared libraries of real tags from genomic DNA, as shown in Figure 1a. The *Mbo*I-digested genomic DNA was size-fractionated (30–60 bp) and subjected to concatemerization, followed by construction of a 2nd library, which contained approximately 10 real tags per clone (Figure 1b). Nucleotide sequence analysis of the real tags revealed that 85.8% mapped to unique positions, which was consistent with our characterization of virtual tags (Figure 1c).

### Amplifications on chromosome 12p in HSC45 gastric cancer cells

**Figure 2:**
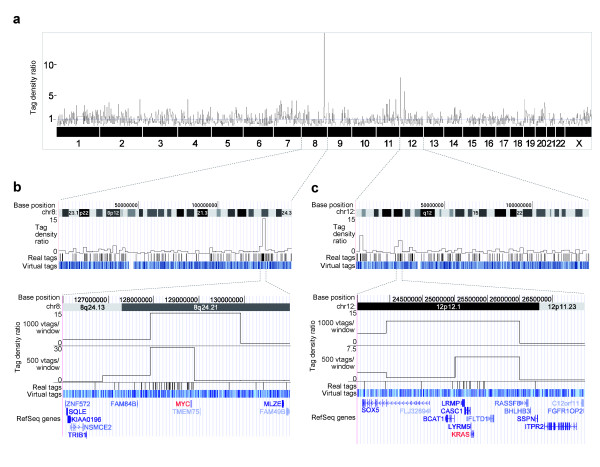
**Detection of increased copy number on chromosomes 8q and 12p by DGS in HSC45 gastric cancer cells**. (**a**) A whole-genome view of the tag density ratio (using a window of 1000 virtual tags) in HSC45 cells as determined by DGS. Values on the y-axis indicate fold-changes in tag density relative to the average tag density of the whole genome, and represent DNA content per haploid genome, in windows. The x-axis represents chromosome number. (**b **and **c**) Expanded view of tag density ratios on chromosomes 8 (**b**) and 12 (**c**). In each panel, the upper graph shows a whole-chromosome view of the tag density ratio (based on a window of 1000 virtual tags). The lower graph shows an expanded view of 8q24.21 and 12p12.1, in which increased tag density was detected using windows of 1000 and 500 virtual tags. Unique real tags are indicated as black vertical bars, and unique virtual tags are indicated in blue (60 bp or shorter) or light blue (longer than 60 bp) bars in dense mode. The positions of refseq genes, with some splicing isoforms omitted, are shown at the bottom of the lower panels.

The genome-wide tag density profile of HSC45 cells was determined using a total of 5,462 unique real tags. To achieve high resolution and sensitivity with the experimental data, we used window sizes of 1000 and 2100 virtual tags (approximately 2300 kb and 4700 kb) for the analysis of amplifications and deletions, respectively. The tag density ratio was calculated as the sum of real tags divided by the average number of real tags in same-sized windows throughout the genome, in which the normal tag density ratio was defined as 1.0. We identified distinct subchromosomal regions of increased tag density at 8q24.21, 12p12.1 and 12p13.33, and decreased tag density at 9p21.3 and the long arm of chromosome 18 (Figure 2, Additional file 9a-d). The regions of increased tag density (12p12.1, 12p13.33 and 8q24.21) contained *KRAS*, *CACNA1C *(calcium channel, voltage-dependent, l type, alpha-1c subunit) and *MYC *loci, respectively. Southern blot analysis confirmed that *KRAS *and *MYC *were amplified in HSC45 cells (Additional file 9e). Each quantitated copy-number change as determined from quantitative real-time PCR (qPCR) of genomic DNA was remarkably similar to that estimated by DGS when the window size for tag density analysis was matched to the size of each alteration (Additional file 9a-d). These results suggest that tag density analysis by DGS could be used to perform copy number analysis throughout the human genome.

### Amplification of *KRAS *in gastric cancer cell lines

**Figure 3:**
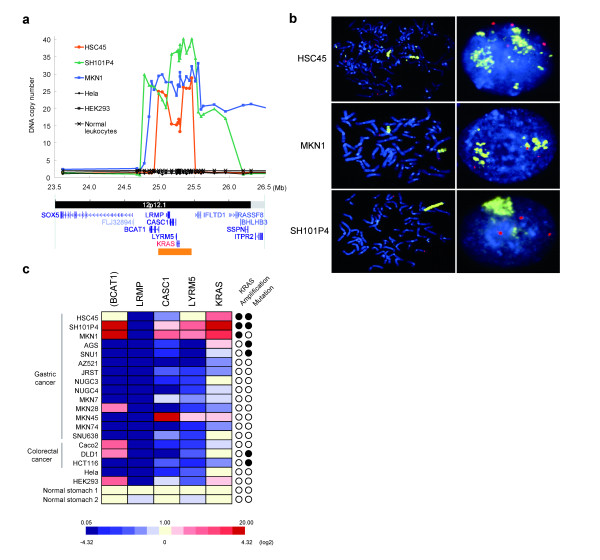
**Gene amplification of *KRAS *in gastric cancer cells**. (**a**) Quantitative genomic PCR analysis of the *KRAS *locus at 12p12.1 in HSC45 cells. Discrete amplifications at 12p12.1 in two other gastric cancer cell lines were also detected (SH101P4 and MKN1). DNA copy number relative to normal diploid leukocyte DNA was plotted against chromosomal nucleotide position (in megabases). The positions of refseq genes in the corresponding regions are shown in the bottom map. The minimum amplification region common to all 3 gastric cancer cell lines is represented by the orange-colored bar. (**b**) Metaphase (left)- and interphase (right)-FISH analysis of the amplified *KRAS *locus in gastric cancer cell lines. The *KRAS*-specific probe is in yellow, and the control probe, specific for the long arm of chromosome 12, is in red. Tetraploidy in HSC45 and triploidy in SH101P4 and MKN1 cells were observed. (**c**) Quantitative real-time RT-PCR analysis of *KRAS *mRNA expression in gastric cancer cells with 12p12.1 amplification. Expression analysis of genes (*KRAS, LRMP, CASC1 *and *LYRM5*) located within the minimal amplicon, and *BCAT1*, which flanks the minimal amplicon, was performed using real-time RT-PCR. Expression levels were normalized to *GAPDH *mRNA, and are depicted as a color gradient, relative to normal stomach. The gene amplification and mutation (codon 12 or 13) status of *KRAS *for each sample is summarized in the right two columns. Filled circles indicate the presence of amplification or mutation of *KRAS*, and open circles indicate no amplification or no mutation of *KRAS*.

Analysis of 26 loci within and immediately flanking chromosome 12p12.1 in HSC45 cells by qPCR demonstrated that a region of approximately 500 kb, which included the *KRAS *gene locus, was amplified (8-fold amplification, Figure 3a). Genomic qPCR screening detected *KRAS *amplification in two additional gastric cancer cell lines, SH101P4 (18-fold) and MKN1 (13-fold) (Figure 3a), whereas we did not detect amplification of greater than 4-fold in 17 other gastric cancer cell lines, or in 10 colon cancer and 11 pancreatic cancer cell lines (listed in Additional file 1, data not shown). DGS also detected amplification of the *KRAS *locus in MKN1 cells (Additional file 10). The neighboring genes of *KRAS *in the minimal amplicon were *LRMP *(lymphoid-restricted membrane protein), *CASC1 *(cancer susceptibility candidate 1) and *LYRM5 *(LYR motif containing 5). *BCAT1 *(branched chain aminotransferase 1, cytosolic) was also amplified in SH101P4 and MKN1 cells, but not in HSC45 cells. We confirmed that *CACNA1C *was amplified in HSC45 cells, but not in the other gastric, colon, or pancreatic cancer cell lines using genomic qPCR analysis (Additional file 9b; data not shown). Neither *NRAS*, *HRAS *nor *BRAF *amplifications were detected in the above cancer cell lines by genomic qPCR analysis (data not shown). The amplification of *KRAS *was also verified by dual color FISH analysis, in which the *KRAS *amplicon was evident as a homogeneously-stained region in HSC45, SH101P4 and MKN1 cells (Figure 3b).

Sequence analysis of *KRAS *(Additional file 11a) showed that both HSC45 and SH101P4 cells harbored a mutation in codon 12 that resulted in a single amino acid substitution in KRAS (ggt→gTt, G12V), whereas MKN1 cells lacked *KRAS *mutations. The presence of *KRAS *mutations in AGS (G12D), SNU1 (G12D), DLD1 (G13D) and HCT116 (G13D) cells has been reported previously [28,29]. Of the ten PCR-clones of *KRAS *from HSC45 and SH101P4 cells that were subjected to mutational analysis, eight and three, respectively, harbored mutations in codon 12. Furthermore, genomic real-time PCR analysis using probes that were specific to wild-type and mutant *KRAS *alleles (Additional file 11b) also revealed that HSC45 and SH101P4 cells contain different proportions of the mutant allele (80% and 50%, respectively). Overall, these results indicated that amplification of a mutant *KRAS *allele also occurs in HSC45 and SH101P4 cells.

**Figure 4:**
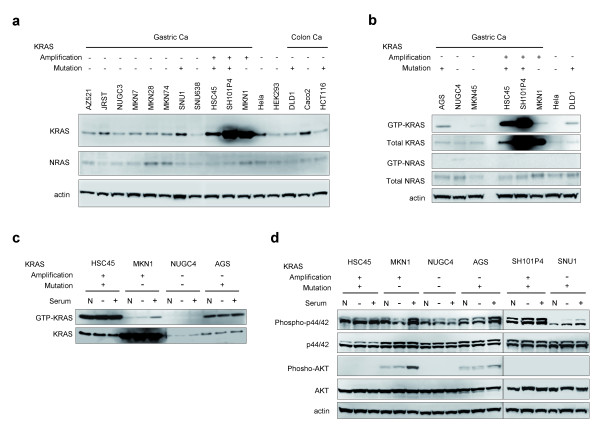
**Overexpression of KRAS, and differential activation of KRAS, p44/42 MAP kinase and AKT in *KRAS*-amplified gastric cancer cells**. (**a**) Immunoblot analysis of the expression levels of KRAS and NRAS in cancer cells. Actin expression was analyzed as a loading control. (**b**) The basal level of GTP-KRAS was markedly high in gastric cancer cells with amplified mutant *KRAS *(HSC45 and SH101P4). Total lysate (500 μg) was subjected to a GTP-RAS pull-down assay, and GTP-KRAS and GTP-NRAS were detected by immunoblot using anti-KRAS and anti-NRAS antibodies, respectively. Total cell lysate (50 μg) was analyzed in parallel to determine the level of expression of KRAS and NRAS in cells. (**c**) GTP-KRAS was elevated after serum stimulation in MKN1 cells. Cells were cultured in regular medium containing 10% FCS (N), serum-starved for 24 h (-) or serum-starved then stimulated with 10% FCS for 1 h (+). Total cell lysate was subjected to a GTP-KRAS pull-down assay. (**d**) Activation of p44/42 MAP kinase and AKT in serum-starved or -stimulated gastric cancer cells. Total cell lysate was analyzed as described for figure **c**. The phosphorylation of p44/42 MAP kinase and AKT was detected by immunoblot using anti-phospho-specific antibodies. In each panel, the status of gene amplification and mutation (codon 12) of *KRAS *in each cell line is indicated. +, presence; -, absence.

We next investigated the levels of *KRAS *mRNA in *KRAS*-amplified gastric cancer cells by quantitative real-time RT-PCR (qRT-PCR) (Figure 3c). The levels of *KRAS *mRNA correlated significantly with *KRAS *copy number. The neighboring genes *LYRM5 *and *CASC1*, which localized to the minimal amplicon, were also expressed at higher levels in cells with amplification as compared to cells without amplification (Figure 3c). Interestingly, *LRMP *was down-regulated in cancer cells as compared to normal stomach cells. Immunoblot analysis of RAS proteins (Figure 4a) revealed that the expression of KRAS was increased in *KRAS*-amplified gastric cancer cells (HSC45, SH101P4 and MKN1), while neither NRAS nor HRAS were highly expressed (Figure 4a; data not shown). Although the expression of let-7c and let-7g microRNAs has been reported to regulate RAS expression [8], we found little correlation of expression of these microRNAs with KRAS protein levels (Additional file 12), which suggested that KRAS overexpression in gastric cancer cell lines is due primarily to genomic amplification of *KRAS*.

### Activation of downstream signaling in *KRAS*-amplified gastric cancer cells

To investigate KRAS activity in gastric cancer cells, we analyzed the amount of GTP-KRAS in cells using an *in vitro *pull-down assay. There was a higher amount of GTP-KRAS in HSC45 and SH101P4 cells, which carried amplified mutant *KRAS*, than in MKN1 cells, in which the level of GTP-KRAS was comparable to AGS cells, which carried non-amplified mutant *KRAS *(Figure 4b). Serum stimulation had little effect on the level of GTP-KRAS in HSC45 cells, but resulted in a dramatic elevation of GTP-KRAS in MKN1 cells (Figure 4c). As expected, this data was consistent with constitutively active mutant KRAS overexpression due to amplification, and it suggested that overexpression of wild-type KRAS may also promote oncogenic properties when cells are exposed to external stimuli.

To gain further insight into the role of overexpressed KRAS in cancer cell growth, we analyzed the activation of p44/42 MAP kinase and AKT (Figure 4d), which are pivotal molecules in the MAP kinase cascade and PI3K signaling pathways that are downstream of KRAS [3,30]. Under normal culture conditions (Figure 4d, lanes indicated as "N"), basal phosphorylation of p44/42 was increased in *KRAS*-amplified cells (HSC45, MKN1, and SH101P4) as compared to NUGC4 gastric cancer cells, in which there is neither amplification nor mutation of *KRAS*. While the phosphorylation of p44/42 was modestly increased in MKN1 cells after serum stimulation, the effect of serum stimulation on HSC45 and SH101P4 cells was minimal, which indicated that p44/42 is constitutively active in the latter two cell lines.

**Figure 5:**
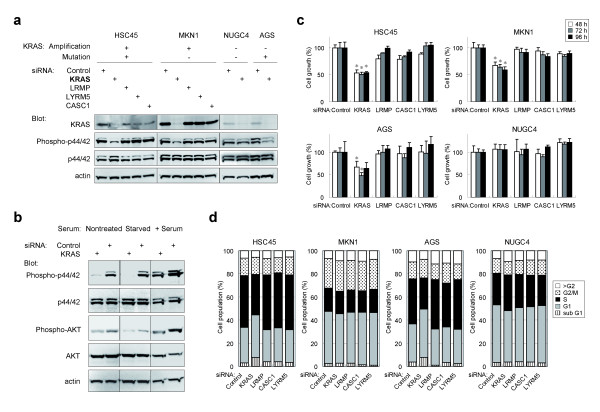
**Suppression of downstream signaling and cell growth in *KRAS*-amplified gastric cancer cells by siRNA-mediated knock-down of *KRAS***. Cells were transfected with siRNA targeting *KRAS, LRMP, CASC1 *or *LYRM5*, or a non-targeting siRNA as a negative control. (**a**) *KRAS *knock-down suppressed the phosphorylation of p44/42 in *KRAS*-amplified cells. Protein levels of KRAS and activated p44/42 48 h post-transfection were determined by immunoblot using anti-KRAS and anti-phospho-p44/42 antibodies. The status of gene amplification and mutation (codon 12) of *KRAS *in each cell line is indicated. +, presence; -, absence. (**b**) *KRAS *knock-down suppressed the phosphorylation of p44/42 MAP kinase and AKT in MKN1 cells. Twenty-four h after siRNA transfection, MKN1 cells were cultured for an additional 24 h in regular medium (Nontreated), serum-starved for 24 h (Starved) or serum-starved then stimulated with 10% FCS for 1 h (+Serum). The activation of p44/42 and AKT was determined by immunoblot using phospho-specific antibodies. (**c**) Suppression of cell growth in *KRAS*-amplified cells by *KRAS *knock-down. Cells were transfected with siRNA, and cell number at the indicated time points after transfection was determined indirectly by WST-8 colorimetric assay. Data is presented as percent decrease in cell number as compared to cells transfected with control siRNA at each time point, and represents the means and SD for triplicate cultures. Statistical analysis was performed using the unpaired t-test. *, *P *< 0.005 relative to the siRNA control. Data is representative of two independent assays. (**d**) *KRAS *knock-down decreased the fraction of HSC45 and AGS cells in S-phase. Cells were analyzed by flow cytometry 48 h post-transfection. Data is representative of two independent assays.

To investigate the biological significance of *KRAS *amplification in gastric cancer, we used small interfering RNA (siRNA) to knock-down the expression of *KRAS *or *KRAS *neighboring genes in four gastric cancer cell lines, HSC45 (carrying amplification and mutation of *KRAS*), MKN1 (amplification but no mutation of *KRAS*), AGS (mutation but no amplification of *KRAS*) and NUGC4 (no amplification or mutation of *KRAS*). Knock-down of *KRAS *and three neighboring genes was verified by qRT-PCR (Additional file 13) and KRAS immunoblot analysis (Figure 5a). While the knock-down of *KRAS *in HSC45 and MKN1 cells caused a marked reduction in phosphorylation of p44/42, knock-down of neighboring genes had no effect (Figure 5a, b). Phosphorylation of p44/42 was reduced in *KRAS *knock-down AGS cells, but not in NUGC4 cells. These results indicated that *KRAS *amplification is associated with both transient and constitutive activation of p44/42 MAP kinase.

Basal phosphorylation of AKT was detected under normal culture conditions, and was increased after serum stimulation of MKN1 and AGS cells (Figure 4d). Nucleotide sequence analysis revealed a single nucleotide mutation at codon 545 of *PIK3CA *in MKN1 and AGS cells (E545K and E545A, respectively, Additional file 11c), which suggested that AKT is potentially activated in these cells through mutational activation of PIK3CA. However, in MKN1 cells, phosphorylation of AKT was reduced by *KRAS *knock-down under both the normal culture condition as well as after serum stimulation (Figure 5b), which suggested that the overexpression of wild-type KRAS might also be involved in enhancing the activation of AKT.

### Growth inhibition of gastric cancer cells with amplification at 12p12.1 by the downregulation of KRAS

Among the four genes that localized to the minimal amplicon at 12p12.1, the knock-down of *KRAS *caused a significant inhibition of cell growth in HSC45, MKN1 and AGS cells (Figure 5c, *P *< 0.005, t-test), whereas knock-down of the other three genes had no effect. There was little growth inhibition observed in NUGC4 cells, in which *KRAS *is neither amplified nor mutated. These results suggested that *KRAS *is the driver gene responsible for the promotion of proliferation of cancer cells harboring 12p12.1 amplification, and the other three genes are likely to be passenger genes on the amplicon. *KRAS *knock-down HSC45 and AGS cells exhibited a reduced accumulation of S-phase cells (Figure 5d), whereas *KRAS *knock-down had no effect on S-phase accumulation in MKN1 cells. Taken together, these results suggested that the amplification of *KRAS *is involved in the promotion of cancer cell growth through the activation of the p44/42 MAP kinase pathway, and in part through activation of the AKT pathway.

### Amplification of wild-type *KRAS *in primary gastric cancer

**Figure 6:**
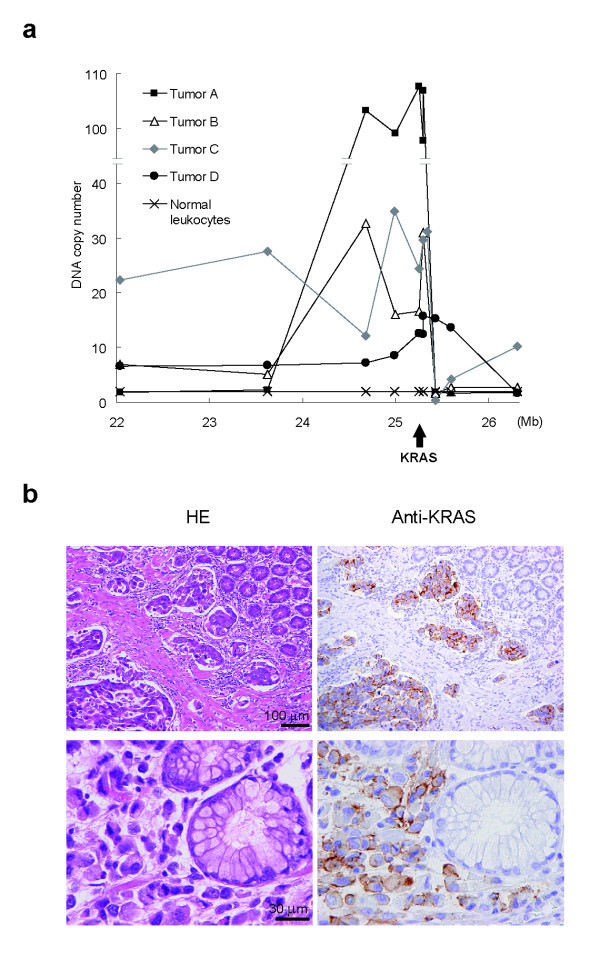
**Amplification of *KRAS *in primary gastric cancer**. (**a**) Quantitative PCR analysis of genomic DNA from primary gastric cancer specimens was carried out using primers specific for regions within and flanking the *KRAS *locus. DNA copy number relative to normal diploid leukocyte DNA is plotted onto the corresponding chromosomal nucleotide position in megabases. (**b**) KRAS is preferentially expressed in gastric cancer cells with *KRAS *amplification. Hematoxylin-Eosin staining (left) and immunohistochemical staining with an anti-KRAS antibody (right) of gastric cancer tissue (tumor D). Upper panels: poorly-differentiated adenocarcinoma cells with submucosal invasion. Lower panels: adenocarcinoma cells adjacent to normal pyloric glands.

To determine the role of *KRAS *in primary gastric cancer, we used qPCR to analyze *KRAS *amplification in genomic DNA derived from primary gastric cancer specimens. We screened 86 specimens, and found amplification of the *KRAS *locus (8–50-fold) in four of them (4.7%) (Figure 6a). Furthermore, mutations were not detected in *KRAS *or *PIK3CA *(exon 9 and 20) in these four tumors by nucleotide sequencing analysis of the PCR products or clones of the PCR products (data not shown). With the exception of tumor C, histopathology of the tumors indicated that they were generally of the diffuse-type, according to the Lauren's classification system [22], and there were no significant differences in clinicopathological features between *KRAS*-amplification-positive and -negative tumors (Additional file 2). Immunohistochemical analysis specifically detected KRAS in cancer cells (tumor D, Figure 6b), whereas the expression of KRAS in adjacent noncancerous cells was below the level of detection. Gene amplification coincided with intense KRAS immunoreactivity in the same tumor samples, which suggested that gene amplification results in the overexpression of the KRAS in primary gastric cancer.

## Discussion

In this report, we described a novel method, termed DGS, of detecting copy number alterations in the human genome, which is based on the analysis of short fragments of genomic DNA generated by restriction enzyme digestion. Although DGS is modeled on the basic concept of DK, we developed a modified tag preparation technique that involves single restriction enzyme digestion without PCR to minimize complex handling regimes and potential biases generated by PCR. Our relatively small-scale sequencing of approximately 5000 tags successfully detected discrete 500-kb amplifications of *KRAS *and *CACNA1C *in HSC45 cells, which were not previously reported in an independent experiment using BAC-based aCGH analysis [31].

To date, however, this DGS method has some limitations as compared to DK and other methods. First, the resolution of DGS using short *Mbo*I tags is lower than DK due to the difference of the theoretical number of virtual tags produced by restriction digestion. The number of virtual tags in our analysis (approximately 394,000 virtual tags in the range of 30 to 60 bp) was less than that of DK (approximately 731,000 tags) [14]. Thus, while the current pilot study demonstrates the feasibility of using DGS to estimate copy number using a simplified tag preparation method, additional studies are needed, using different or combinations of restriction enzymes to produce more short tags, to improve the resolution of DGS. Second, DGS method has several limitations involved in labor, cost, and amount of material: (a) this method needs the generation of two rounds of plasmid libraries and the propagation of plasmid libraries, (b) this method costs higher than microarray platform and DK, (c) a large amount of starting material DNA is required.

Recently, the use of single nucleotide polymorphism (SNP) arrays for the detection of allele-specific copy-number alterations at high resolution using 906,600 SNP probes has been reported [32]. Because DGS and DK do not rely on pre-designed probes, they are "open" platform techniques. For example, DK could be used to explore exogenous pathogenic DNA in infectious or neoplastic states [14]. However, tag-counting methods, including DGS and DK, have similar limitations. First, they generally do not estimate allele-specific copy number, which SNP array analysis does. Second, the number of sequence reads, which is to say, the depth of sequencing, affects the sensitivity and the resolution of tag density profiles. The results of simulated DGS indicated that DGS using deep sequencing will have a higher level of sensitivity in detecting subtle copy-number alterations. However, even in reports of successful DK [14-17], the depth of sequencing was less than 0.3 (when the theoretical number of unique virtual tags was defined as 1.0), partly due to practical limitations, such as the low through-put rate and labor intensive methods required when using standard sequencers [33]. In the next step of improving DGS, DGS should be combined with the next-generation sequencing technologies [34]. The recent introduction of instruments capable of sequence millions of nucleotides in a single run is changing the landscape of human genetics. By applying next-generation sequencing technologies to DGS, it should be possible to simplify the protocol and improve efficiency and resolution by bypassing the multi-step process of tag concatemerization, as well as conserve starting genomic DNA. With some next-generation sequencers, tag preparation by restriction digestion might generate more reproducible DNA fragmentation than current random-shearing approaches [35,36].

Gene amplification of *KRAS *with or without mutation has been described in a limited number of cases, including lung, gastric, pancreatic and rectal cancers [37-40]. More recently, aCGH analysis of various primary tumors, including lung, colorectal, pancreatic and gastric cancers, gliomas and testicular germ cell tumors, also detected amplification of chromosome 12p [41-46]. In this report, we provided evidence that, while rare in colon and pancreatic cancers, the incidence of *KRAS *gene amplification (greater than 4-fold) is increased in gastric cancer, and is responsible for KRAS activation.

Using MKN1 cells as a model system, we investigated the mechanism by which *KRAS *amplification contributes to the growth of primary gastric cancers that lack mutations in *KRAS*. Immunoblot analysis and knock-down of *KRAS *in cells provided evidence that *KRAS *gene amplification results in KRAS activation in the absence of mutation. To our knowledge, this is the first report to demonstrate a potential relationship between gene amplification of endogenous wild-type *KRAS*, activation of KRAS signaling pathways, and cell growth in gastric cancer. In general, less than 10% of wild-type and over 50% of mutant RAS is in the GTP-bound state in cells [47,48]. Therefore, it is likely that amplification of endogenous wild-type *KRAS *coupled overexpression in the MKN1 cells induces a biological effect that is similar to the effect of single-mutant alleles of *KRAS*. We also found that while serum stimulation induced the activation of overexpressed KRAS and p44/42 in MKN1 cells, in cells that harbored amplified mutant *KRAS*, KRAS and p44/42 were constitutively activated. Thus, amplified wild-type *KRAS *might provide a growth advantage to cancer cells, not only by upregulating the basal cell growth, but also by conferring adaptability to changes in the environment, such as availability of growth factors and nutrients. Further studies will be needed to investigate potential functional connections for these correlations.

The *KRAS *gene status of tumors is currently of great interest, because *KRAS *mutations are linked to the response to anti-epidermal growth factor receptor (EGFR) therapies. Panitumumab and cetuximab are antibody-based drugs that inhibit EGFR, and are currently used in the treatment of colorectal cancer [49]. However, several groups have reported that *KRAS *mutations are significantly associated with lack of response to cetuximab or panitumumab in patients with advanced, chemotherapy-refractive colorectal cancer [50,51]. In gastric cancer, EGFR is a promising target since it is frequently overexpressed [52,53], and clinical trials of cetuximab in the treatment of gastric cancer are ongoing [54,55]. Our results showing that overexpressed wild-type KRAS is involved in the activation of downstream signaling pathways that govern cell proliferation indicate that the amplification of *KRAS *might be of clinical significance in predicting response to cetuximab or to panitumumab in gastric cancer. Prospective studies are needed to determine the efficacy of patient-specific EGFR-targeted therapy based on *KRAS *amplification and mutation status.

## Conclusion

We demonstrated that DGS is an efficient method of identifying DNA copy-number alterations. Using DGS, we investigated the role of *KRAS *gene amplification in the overactivation of KRAS in gastric cancer. Future studies using a larger cohort of gastric cancer specimens are needed to elucidate the clinical, diagnostic and therapeutic significance of *KRAS *amplification and overexpression.

## Competing interests

The authors declare that they have no competing interests.

## Authors' contributions

HM performed molecular biological experiments including DGS, and wrote Perl scripts and the paper. FA, HA, RM and HT performed *in silico *genome analyses and constructed the tag database. YS and MI performed real-time PCR. LK performed Southern blotting. MT and HS extracted genomic DNA and performed sequencing. KY provided gastric cancer cell lines. MF, MH and MK provided primary samples and clinico-pathological data. SVS designed *in silico *DGS simulation and performed statistical analyses. KI, YS and TT conceived, coordinated the study and revised the paper. All authors read and approved the final manuscript.

## Pre-publication history

The pre-publication history for this paper can be accessed here:

http://www.biomedcentral.com/1471-2407/9/198/prepub

## Supplementary Material

Additional file 1 Cell lines used in this study. Click here for file

Additional file 2 Clinicopathological features of primary gastric carcinomas with or without *KRAS *amplification. Click here for file

Additional file 3 Additional Methods
Characterization of virtual tags and construction of virtual tag database
DGS simulation *in silico*
DGS: preparation of real tag library, tag sequencing and tag density analysis
Southern blot analysis
Quantitative evaluation of mutant alleles of *KRAS* miR RT-PCR. Click here for file

Additional file 4 Primers for genomic qPCR. Click here for file

Additional file 5 Gene Expression Assay number and primer/probe sequences for qRT-PCR. Click here for file

Additional file 6 Primers for mutational analysis. Click here for file

Additional file 7 **Characteristics of *Mbo*I virtual tags**. (**a**) Repeat and (**b**) Uniqueness classification of *Mbo*I virtual tags. The actual number of *Mbo*I virtual tags (left) and the corresponding proportion (right) of tags of each length (from 20 bp to 130 bp) are shown. (**c**) Summary of the Repeat/Uniqueness classification of *Mbo*I virtual tags. (**d-f**) Frequency of intervals between two unique virtual tags in the range of 30 to 60 bp (**d**), in the range of 70 to 100 bp (**e**), and in the range of 100 to 130 bp (**f**). The interval frequency, in 200 bp, for each range is plotted. Corresponding cumulative frequency is also shown in each plot. (**g**) Chromosomal distribution of unique virtual tags. The actual number of unique virtual tags and the corresponding proportion in each chromosome are shown. Click here for file

Additional file 8 Theoretical detection of copy number alteration by DGS. Click here for file

Additional file 9 **Genome regions with copy number alterations in HSC45 cells, as detected by DGS**. (**a–d**) DGS identified amplifications at 8q24.2 (**a**) and 12p13.33 (**b**), which contain *MYC *and *CACNA1C*, respectively; a deletion at 9p21.3, which contains *CDKN2A *(**c**); and a copy number decrease at the long arm of chromosome 18 (**d**) in HSC45 cells. The upper panel of each figure shows the tag density ratio, the maps of real and virtual tags, and refseq genes. The lower panel shows genomic qPCR analysis of copy number. DNA copy number was normalized to Line-1, a repetitive element, and normal diploid leukocyte DNA. (**e**) Gene amplification of *KRAS *and *MYC *in HSC45 gastric cancer cells was confirmed by Southern blot analysis. The indicated amounts of genomic DNA from HSC45 and HEK293 cells were digested with *Msp*I, separated by 0.8% agarose gel electrophoresis, and then analyzed by Southern blot using *KRAS*- and *MYC*-specific probes. Click here for file

Additional file 10 **Amplification of the chromosomal region from 12p12.1 to 12p11.22, which includes the *KRAS *locus, was detected in MKN1 gastric cancer cells by DGS**. (**a**) Whole-genome profile of the tag density ratio (determined using a window of 1000 virtual tags) of MKN1 cells. (**b**) Whole-chromosome view of the tag density ratio (using a window of 3000 virtual tags) of chromosome 12. Unique real tags are indicated as black vertical bars in squish mode, and unique virtual tags are indicated in blue (60 bp or shorter) or light blue (longer than 60 bp) bars in dense mode. The position of the *KRAS *locus is indicated at the bottom. Click here for file

Additional file 11 **Missense mutations of *KRAS *and *PIK3CA*, and amplified mutant alleles of *KRAS *in gastric cancer cells**. (**a**) Mutation of codon 12 of *KRAS *in HSC45, SH101P4 and AGS cells. Sequence chromatograms of *KRAS *missense mutations were generated by nucleotide sequencing of PCR products directly, or sequencing of PCR clones. Mutated codons are underlined. Representative results from PCR clones are shown. (**b**) Amplified mutant alleles of *KRAS *in HSC45 and SH101P4 cells. The allelic proportion of mutant *KRAS *(G12V, ggt→gTt) was analyzed by duplex real-time PCR using mutant (gTt) and wild-type (ggt) allele-specific probes labeled by FAM and VIC, respectively. Serial dilutions of vectors for mutant (M) or wild-type (W) *KRAS *were mixed at the indicated ratios, and then used as standards. The fluorescence intensity of the two different dyes is presented as a two-dimensional plot. (**c**) Mutations of codon 545 of *PIK3CA *in MKN1 and AGS cells. Mutated codons are underlined. Representative results from cloned PCR products are shown. Click here for file

Additional file 12 **Expression of the microRNAs let7-c and let7-g in gastric cancer cells that overexpress KRAS**. Semiquantitative RT-PCR analysis of microRNAs was carried out using small RNAs derived from the indicated cell lines. The expression levels of let7-a, U6 and hsa-miR-24 were analyzed as controls. Reaction products were analyzed by 3.0% Nusieve agarose gel electrophoresis. Click here for file

Additional file 13 **Gene expression in siRNA knock-down cells**. Gastric cancer cell lines were transfected with siRNAs for *KRAS*, *LRMP*, *CASC1*, *LYRM5*, or a universal non-targeting siRNA as a negative control. Cells were cultured for 48 h and then total RNA was isolated. mRNA expression of *KRAS *(**a**) and *LRMP*, *CASC1 *and *LYRM5 *(**b**) in each cell line was determined by qRT-PCR. The expression of each gene was normalized to that of *GAPDH *and normal stomach mRNA. Non, nontransfected cells. Data represents the means and SD of three independent experiments. Click here for file
