# Supplementary material for: A novel method, digital genome scanning detects KRAS gene amplification in gastric cancers: involvement of overexpressed wild-type KRAS in downstream signaling and cancer cell growth
Source: BMC Cancer. 2009 Jun 23;9:198. doi: 10.1186/1471-2407-9-198 (PMC2717977; doi:10.1186/1471-2407-9-198)
Supplement: Additional file 2 — Clinicopathological features of primary gastric carcinomas with or without KRAS amplification. [file 1471-2407-9-198-S2.pdf]

Additional file 2. Clinicopathological features of primary gastric carcinomas with or without *KRAS* amplification

Characteristics	No. of patients (%)			*P*
	Total	*KRAS* amplification (+)	*KRAS* amplification (-)	
No. of patients	86	4	82	
Age (yrs)				0.5086
Median	66	60.5	67	
Range	36-88	51-70	36-88	
Sex				1.0000
Male	58 (67.4)	3 (75.0)	55 (67.1)	
Female	28 (32.6)	1 (25.0)	27 (32.9)	
Histology (Lauren)				0.6261
Intestinal	39 (45.3)	1 (25.0)	38 (46.3)	
Diffuse	47 (54.7)	3 (75.0)	44 (53.7)	
Histopathological grade				0.5926
Well differentiated	16 (18.6)	1 (25.0)	15 (18.3)	
Moderately differentiated	23 (26.7)	0 (0.0)	23 (28.0)	
Poorly differentiated	47 (54.7)	3 (75.0)	44 (53.7)	
Pathological primary tumor status				0.0803
pT1	9 (10.5)	1 (25.0)	8 (9.8)	
pT2	42 (48.8)	3 (75.0)	39 (47.6)	
pT3	33 (38.4)	0 (0.0)	33 (40.2)	
pT4	2 (2.3)	0 (0.0)	2 (2.4)	
Pathological lymph node status				0.3996
pN0	23 (26.7)	1 (25.0)	22 (26.8)	
pN1	23 (26.7)	0 (0.0)	23 (28.0)	
pN2	28 (32.6)	2 (50.0)	26 (31.7)	
pN3	12 (14.0)	1 (25.0)	11 (13.4)	
Pathological metastasis status				0.5799
pM0	68 (79.1)	4 (100.0)	64 (78.0)	
pM1	18 (20.9)	0 (0.0)	18 (22.0)	
Stage (pTNM)				0.7437
IA	7 (8.1)	1 (25.0)	6 (7.3)	
IB	11 (12.8)	0 (0.0)	11 (13.4)	
II	12 (14.0)	0 (0.0)	12 (14.6)	
IIIA	21 (24.4)	2 (50.0)	19 (23.2)	
IIIB	7 (8.1)	0 (0.0)	7 (8.5)	
IV	28 (32.6)	1 (25.0)	27 (32.9)	
