# Supplementary material for: A novel method, digital genome scanning detects KRAS gene amplification in gastric cancers: involvement of overexpressed wild-type KRAS in downstream signaling and cancer cell growth
Source: BMC Cancer. 2009 Jun 23;9:198. doi: 10.1186/1471-2407-9-198 (PMC2717977; doi:10.1186/1471-2407-9-198)
Supplement: Additional file 3 — Additional Methods Characterization of virtual tags and construction of virtual tag database DGS simulation in silico DGS: preparation of real tag library, tag sequencing and tag density analysis Southern blot analysis Quantitative evaluation of mutant alleles of KRAS miR RT-PCR. [file 1471-2407-9-198-S3.pdf]

Additional file 3.

Additional Methods

Characterization of virtual tags and construction of virtual tag database. Short *Mbo*I restriction fragments, which were computationally extracted from the human genome sequence (Mar. 2006 freeze, hg18, http://genome.ucsc.edu/) within the range of 20 bp to 130 bp (including both 5' and 3' of GATC ends), were collected and termed virtual tags. Virtual tags, which contained sequence of any repeat element (short interspersed nuclear elements (SINE) including ALUs, long interspersed nuclear elements (LINE), long terminal repeat elements (LTR) including retroposons, DNA repeat elements, simple repeats (micro-satellites), low complexity repeats, satellite repeats, RNA repeats including RNA, tRNA, rRNA, snRNA, scRNA, srpRNA, and others) were computationally detected and classified as repeat tags, while the remaining tags were classified as non-repeat tags. The information on repeat elements (RepBase library of repeats, originally provided from Genetic Information Research Institute) was obtained from the UCSC genome browser site. Uniqueness analysis of virtual tags was performed by sequence matching of whole virtual tags in a one-versus-others fashion. Virtual tags for which there were no other virtual tags with the same sequences in both strands were classified as unique tags, while the remaining tags were classified as non-unique tags. Virtual tag database which archived information about sequence, genome position and flags of repeat/non-repeat/unique/non-unique of each virtual tag was constructed by using the MySQL 5.0

database system. Histograms of classified virtual tags were created by searching the virtual tag database.

DGS simulation *in silico*. Performance of DGS to detect copy number alterations was estimated by evaluating the theoretical sensitivity, specificity and positive predictive value using Monte Carlo simulations. For each type of alteration, 100 simulations were performed as follows: either 5 000, 10 000 or 100 000 tags were randomly assigned to 400 000 unique virtual tags (60 bp or shorter) in a genome, which contained a single placed alteration with a predefined size and copy number. Moving windows containing the same number of virtual tags as the simulated alteration were used to calculate tag densities along the genome. For each size of window, the threshold of tag density ratio was defined from 100 simulations of the normal genome (with no alterations): for copy number increase, mean of maximum tag density ratio + 2SD (Ti); for copy number decrease, mean of minimum tag density ratio -2SD (Td). Values of tag density ratio (V) were considered true positives when $V > Ti$, $V = 0$ or $Td > V > 0$ was observed in the window of amplification, homozygous deletion or heterozygous loss, respectively. Tag density ratios of these values outside the alteration were considered false positives.

DGS: preparation of real tag library, tag sequencing and tag density analysis. <u>The first library preparation:</u> For each sample, forty μg of genomic DNA was digested with *Mbo*I (Takara) and

electrophoresed through 3% Nusieve agarose gels, after which fragments shorter than 60 bp were electroeluted from an excised gel slice and then purified by the phenol-chloroform extraction method. Concatemers of real tags were prepared in a 20 μl ligation mixture which contains 150 ng of tags, 40 ng of *Bam*HI-digested pBluescript II SK+ vector (Stratagene) and 10 μl of Mighty Mix DNA ligation solution (Takara), and then incubated at 10°C for 16 h. Electrocompetent cells (DH10B, Takara) were transformed with the purified vectors by using Biorad E.coli pulser and grown on LB-amp plates. A total of ~ 100 000 colonies were scraped off, subjected to automatic plasmid isolation system (PI-100, Kurabo, Japan) followed by RNase treatment, and the purified DNA was stored as the 1st library. <u>The second library preparation:</u> 20 μg of the 1st library DNA was double-digested with *Pst*I and *Spe*I (Takara) to excise the concatemers from vectors and electrophoresed in a 3% Nusieve agarose gel, after which longer fragments (140 bp to 800 bp) were electroeluted from an excised gel slice and then purified by phenol-chloroform method. Concatemers of the concatemers were prepared in a 20 μl ligation mixture which contained 150 ng of concatemers, 100 ng of *Spe*I- and/or *Pst*I-digested pBluescript II SK+ vectors and 10 μl of Mighty Mix DNA ligation solution (Takara), and then incubated at 10°C for 16 h. Electrocompetent cells were transformed with the purified vectors, and spread on LB-amp plates for blue-white color selection. Each white colony that contained 2nd library plasmid was picked and cultured overnight in LB-amp media using a 96-well culture block (Qiagen). Plasmids were purified by using Qiaprep 96 turbo kit (Qiagen). <u>Tag sequencing and tag density</u>

analysis: real tags in the 2nd libray vector were sequenced using the Big Dye terminator v3.1 kit (Applied Biosystems) and Big Dye Xterminator purification kit on ABI3130 Genetic Analyzer (Applied Biosystems) according to the manufacturer's instructions. Real tags sequences flanked by GATC ends were extracted from raw sequences obtained from the 2nd library, by using in-house tag analysis scripts (Active Perl 5.8.8). Possible artificial chimeric tags that contained an internal *Pst*I or *Spe*I site were excluded from further analysis. The real tags which matched to unique virtual tags were mapped to the human genome. The remaining real tags corresponded to sequences matched to non-unique virtual tags, sequences not present in the current genome database release, polymorphisms, or sequencing errors in the tags or in the genome database. Tag densities ratio for sliding windows containing N (100 ~ 13 000) virtual tags were dynamically determined as the sum of real tags divided by the average number of real tags in same sized windows throughout the genome. Copy-number alterations were detected by analysis of tag density ratios using threshold values defined by the DGS simulations.

Southern blot analysis. Genomic DNA digested with the *Msp*I restriction enzyme (Takara), was run on a 0.8% agarose gel and then blotted onto a nylon filter (Hybond N+; Amersham). Probe DNA was prepared by PCR from genomic DNA, and the purified PCR product was labeled with [α-P32] dCTP (Amersham) by random DNA labeling (Random primer DNA labeling kit; Takara). Primer sequences

.

used for probe preparation were: *KRAS*, 5'-CGGTAGTTGTAGGTTCTCTAATG-3' and 5'-GTCGATGGAGGAGTTTGTAAATG-3'; *MYC*, 5'-CCTCCAGTAACTCCTCTTTCTTC-3' and 5'-CTGCCTTCCAGGCATTAATTTC-3'. Prehybridization and hybridization were performed as described elsewhere.

Quantitative evaluation of mutant alleles of *KRAS*. The allelic proportion of mutant *KRAS* (G12V, ggt→gTt) was determined using the ABI PRISM 7000 (Applied Biosystems) by employing a modified real-time PCR procedure according to Itabashi et al [26]. Briefly, each reaction mixture contained 0.5 μM of each primer, 250 nM of each fluorogenic-labeled probe, 25 μl of TaqMan universal PCR master mix (Applied Biosystems) and 100 ng of genomic DNA in a final volume of 50 μl. The plasmids which contained the wild or mutant *KRAS* sequence were obtained from the experiments of the mutational analysis, and used as standard samples. The genomic DNA was subjected to 30 cycles of a two-step PCR consisting of a 15 sec denaturing step at 95°C and a 1 min annealing/extension step at 67°C. TaqMan MGB probes to discriminate between mutant (gTt) and wild-type (ggt) alleles were synthesized by Applied Biosystems: 5'-FAM-ttggagctgTtggcgta-MGB-3' (mutant), 5'-VIC-ttggagctggtggcgta-MGB-3' (wild-type). The Primer set was: 5'-AGGCCTGCTGAAAATGACTGAATATAAACTTGTGGTA-3' and 5'-TTCGTCCACAAAATGATTCTGAATTAGCTGTATCGTC-3'. After PCR, fluorescence intensities

.

(delta Rn) for the two different dyes were measured and presented as a two-dimensional plot.

miR RT-PCR. Small RNA was extracted using mirVana microRNA (miRNA) isolation kit (Ambion) according to manufacturer's instruction. miRNA RT-PCRs were performed by using SuperTaq Polymerase (Ambion) and the mirVana qRT-PCR miRNA detection kit (Ambion) following the manufacturer’s instructions. Reactions contained mirVana qRT-PCR Primer Sets specific for let7-a,-c,-g, and U6 and hsa-miR-24 as controls. The PCR exponential phase was determined in the 16- to 20-cycle range to allow semiquantitative comparisons among miRNAs from identical reactions. The reaction products were analyzed on a 3.0% Nusieve agarose gel stained with ethidium bromide.
