# Supplementary material for: A novel method, digital genome scanning detects KRAS gene amplification in gastric cancers: involvement of overexpressed wild-type KRAS in downstream signaling and cancer cell growth
Source: BMC Cancer. 2009 Jun 23;9:198. doi: 10.1186/1471-2407-9-198 (PMC2717977; doi:10.1186/1471-2407-9-198)

Additional file 7.

a

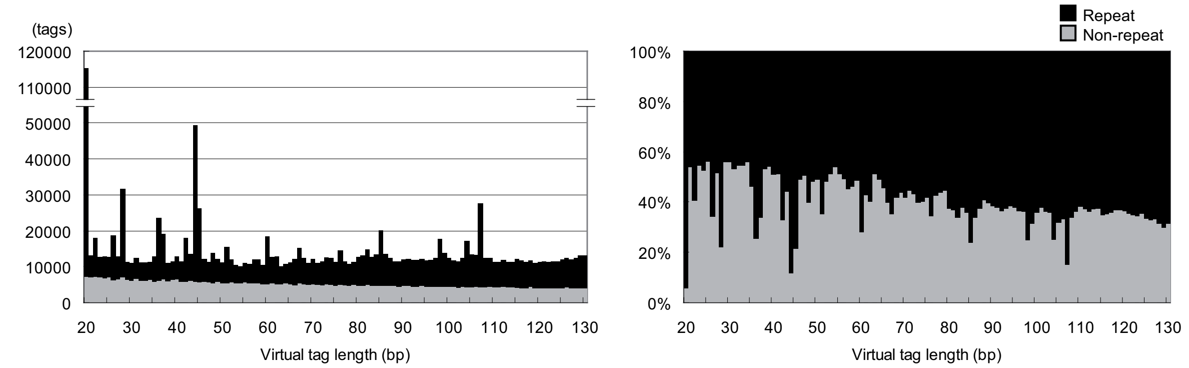

b

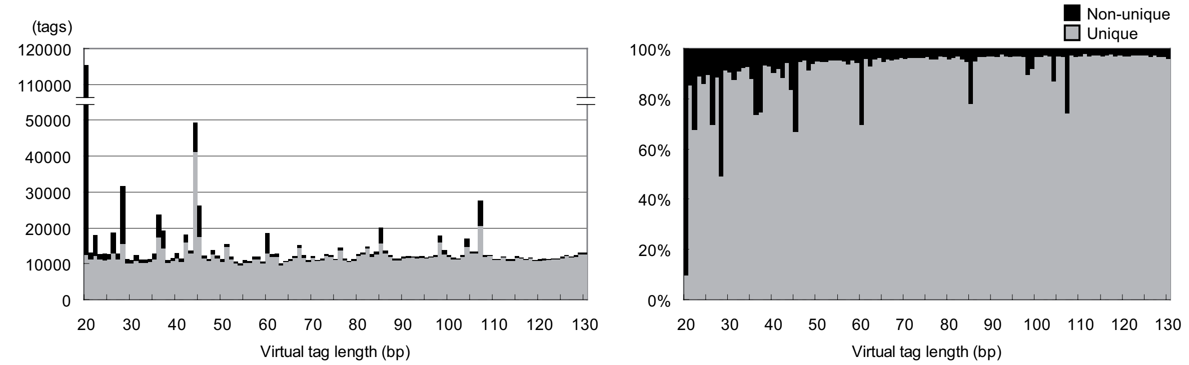

c

20 - 130 bp	tags (%)	Unique	Non-unique	sub total
	Repeat	810 664 (50.7)	224 201 (14.0)	1 034 865 (64.7)
	Non-repeat	540 155 (33.8)	25 229 (1.6)	565 384 (35.3)
	sub total	1 350 819 (84.4)	249 430 (15.6)	1 600 249 (100.0)

30 - 60 bp	tags (%)	Unique	Non-unique	sub total
	Repeat	224 380 (49.8)	45 775 (10.2)	270 155 (60.0)
	Non-repeat	169 941 (37.7)	10 252 (2.3)	180 193 (40.0)
	sub total	394 321 (87.6)	56 027 (12.4)	450 348 (100.0)

d **e** **f**

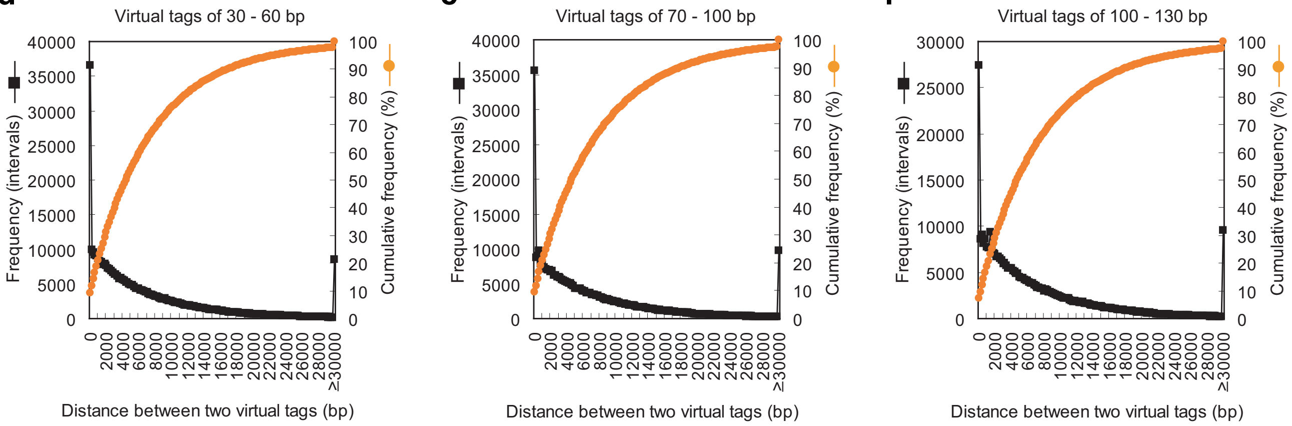

g

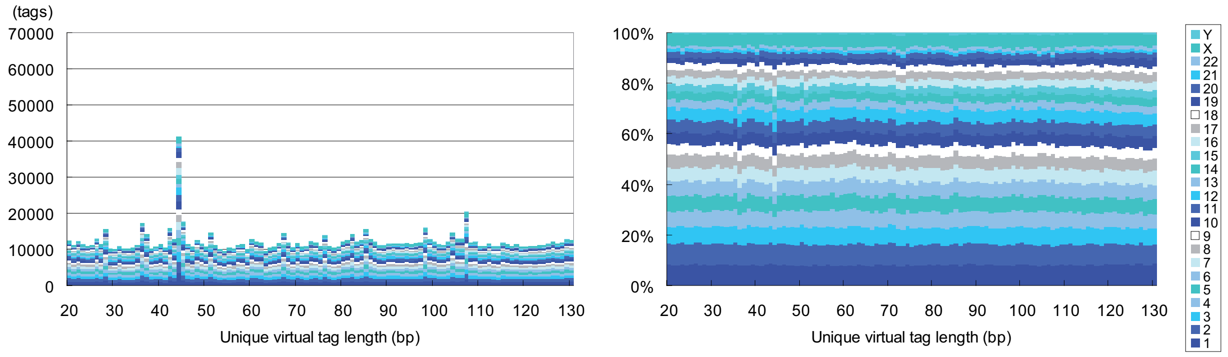

Supplement: Additional file 7 — Characteristics of MboI virtual tags. (a) Repeat and (b) Uniqueness classification of MboI virtual tags. The actual number of MboI virtual tags (left) and the corresponding proportion (right) of tags of each length (from 20 bp to 130 bp) are shown. (c) Summary of the Repeat/Uniqueness classification of MboI virtual tags. (d-f) Frequency of intervals between two unique virtual tags in the range of 30 to 60 bp (d), in the range of 70 to 100 bp (e), and in the range of 100 to 130 bp (f). The interval frequency, in 200 bp, for each range is plotted. Corresponding cumulative frequency is also shown in each plot. (g) Chromosomal distribution of unique virtual tags. The actual number of unique virtual tags and the corresponding proportion in each chromosome are shown. [file 1471-2407-9-198-S7.pdf]
