# Supplementary figures and images for: A novel method, digital genome scanning detects KRAS gene amplification in gastric cancers: involvement of overexpressed wild-type KRAS in downstream signaling and cancer cell growth
Source: BMC Cancer. 2009 Jun 23;9:198. doi: 10.1186/1471-2407-9-198 (PMC2717977; doi:10.1186/1471-2407-9-198)

Additional file 9.

a

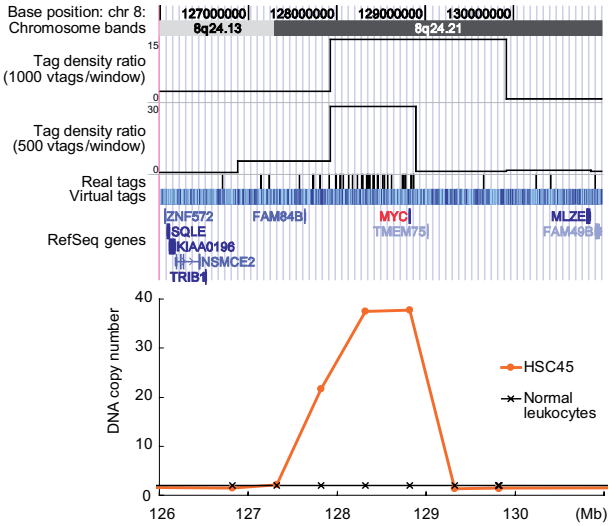

b

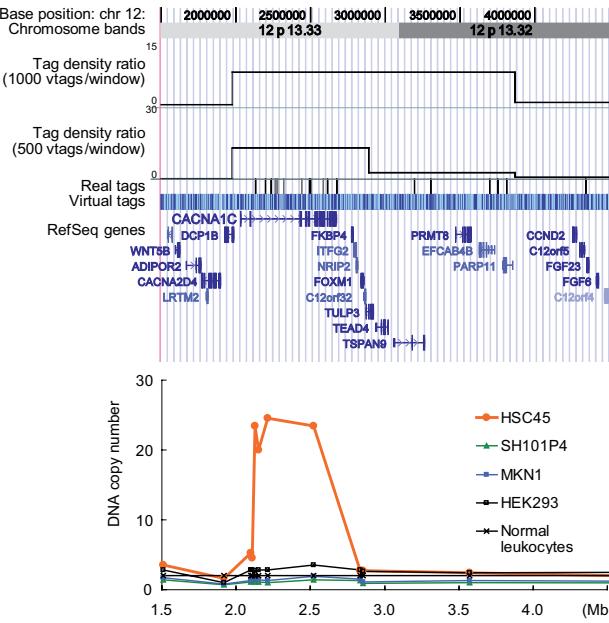

c

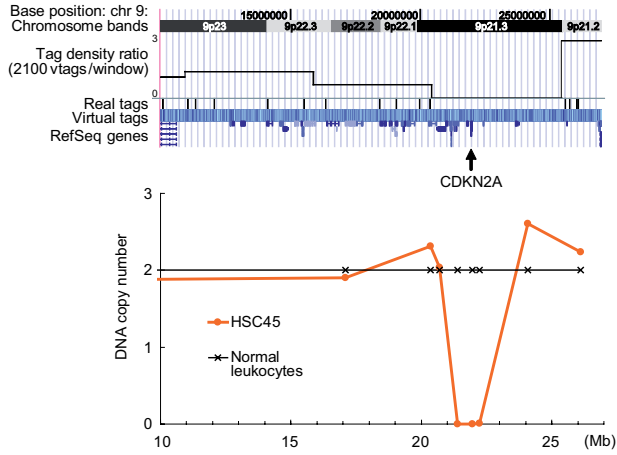

d

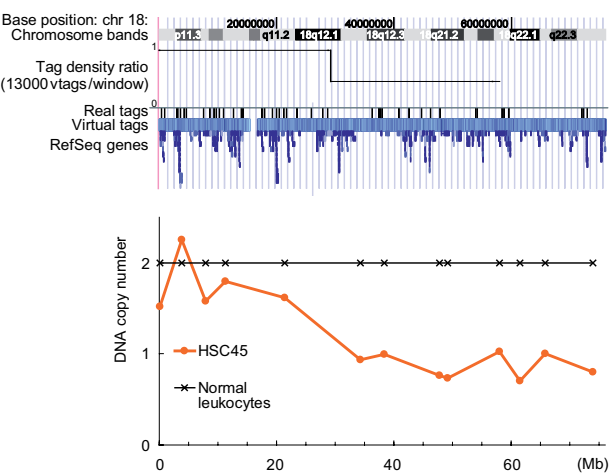

e

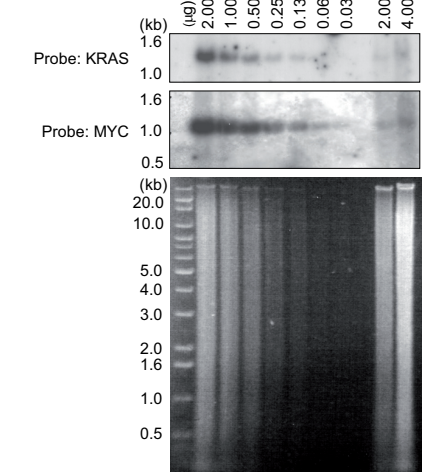

Supplement: Additional file 9 — Genome regions with copy number alterations in HSC45 cells, as detected by DGS. (a–d) DGS identified amplifications at 8q24.2 (a) and 12p13.33 (b), which contain MYC and CACNA1C, respectively; a deletion at 9p21.3, which contains CDKN2A (c); and a copy number decrease at the long arm of chromosome 18 (d) in HSC45 cells. The upper panel of each figure shows the tag density ratio, the maps of real and virtual tags, and refseq genes. The lower panel shows genomic qPCR analysis of copy number. DNA copy number was normalized to Line-1, a repetitive element, and normal diploid leukocyte DNA. (e) Gene amplification of KRAS and MYC in HSC45 gastric cancer cells was confirmed by Southern blot analysis. The indicated amounts of genomic DNA from HSC45 and HEK293 cells were digested with MspI, separated by 0.8% agarose gel electrophoresis, and then analyzed by Southern blot using KRAS- and MYC-specific probes. [file 1471-2407-9-198-S9.pdf]

## Additional file 10.

**a**

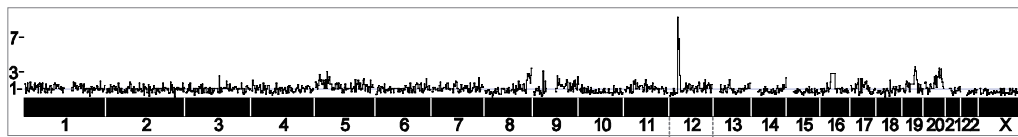

**b**

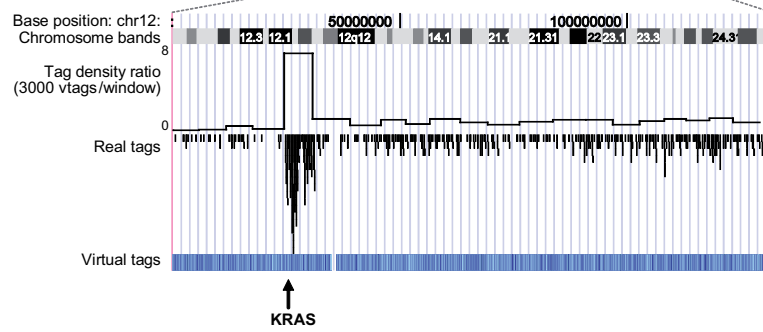

Supplement: Additional file 10 — Amplification of the chromosomal region from 12p12.1 to 12p11.22, which includes the KRAS locus, was detected in MKN1 gastric cancer cells by DGS. (a) Whole-genome profile of the tag density ratio (determined using a window of 1000 virtual tags) of MKN1 cells. (b) Whole-chromosome view of the tag density ratio (using a window of 3000 virtual tags) of chromosome 12. Unique real tags are indicated as black vertical bars in squish mode, and unique virtual tags are indicated in blue (60 bp or shorter) or light blue (longer than 60 bp) bars in dense mode. The position of the KRAS locus is indicated at the bottom. [file 1471-2407-9-198-S10.pdf]

Additional file 11.

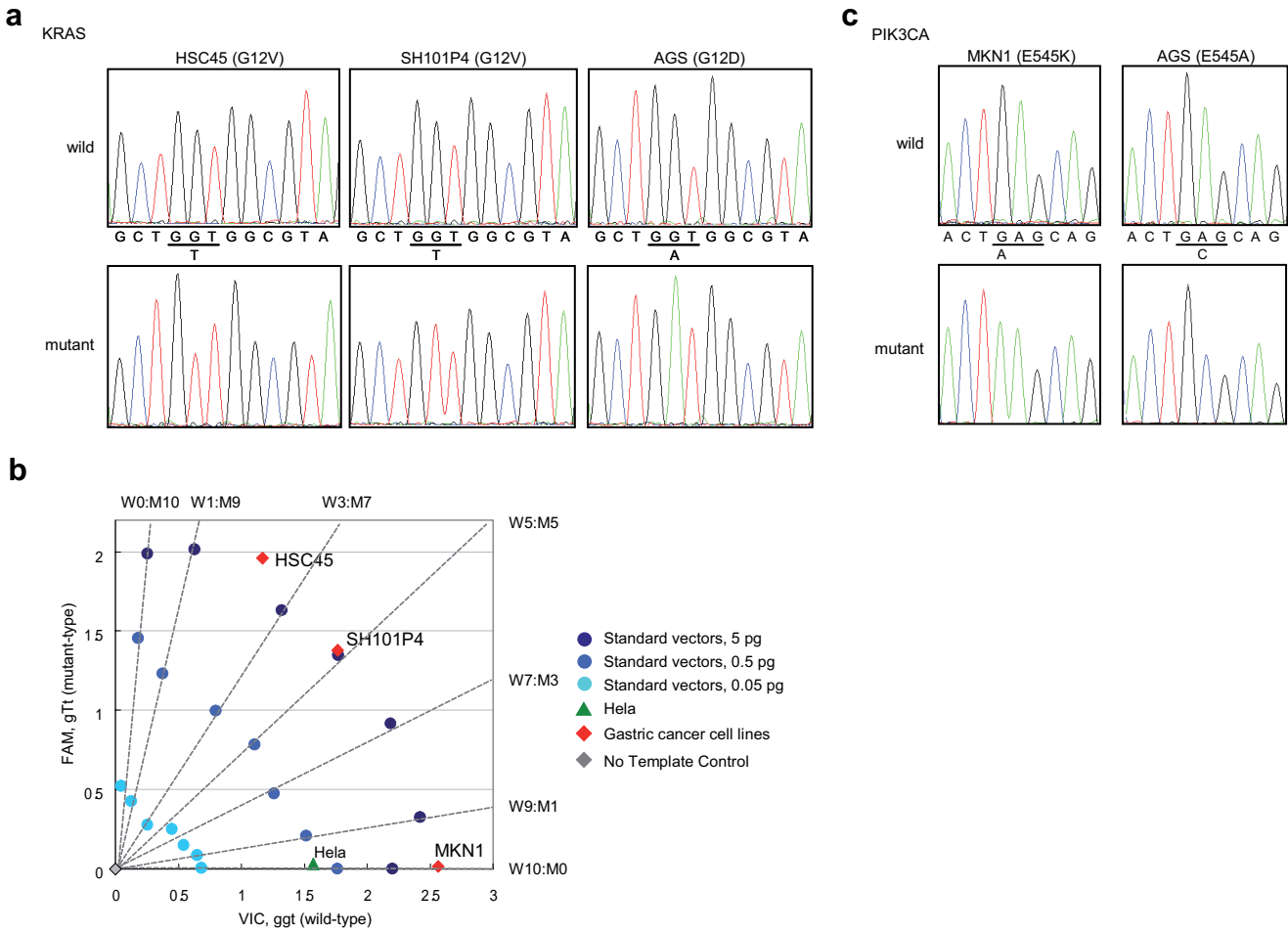

Supplement: Additional file 11 — Missense mutations of KRAS and PIK3CA, and amplified mutant alleles of KRAS in gastric cancer cells. (a) Mutation of codon 12 of KRAS in HSC45, SH101P4 and AGS cells. Sequence chromatograms of KRAS missense mutations were generated by nucleotide sequencing of PCR products directly, or sequencing of PCR clones. Mutated codons are underlined. Representative results from PCR clones are shown. (b) Amplified mutant alleles of KRAS in HSC45 and SH101P4 cells. The allelic proportion of mutant KRAS (G12V, ggt→gTt) was analyzed by duplex real-time PCR using mutant (gTt) and wild-type (ggt) allele-specific probes labeled by FAM and VIC, respectively. Serial dilutions of vectors for mutant (M) or wild-type (W) KRAS were mixed at the indicated ratios, and then used as standards. The fluorescence intensity of the two different dyes is presented as a two-dimensional plot. (c) Mutations of codon 545 of PIK3CA in MKN1 and AGS cells. Mutated codons are underlined. Representative results from cloned PCR products are shown. [file 1471-2407-9-198-S11.pdf]

Additional file 12.

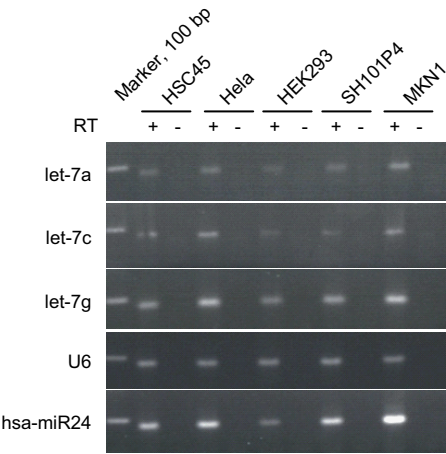

Supplement: Additional file 12 — Expression of the microRNAs let7-c and let7-g in gastric cancer cells that overexpress KRAS. Semiquantitative RT-PCR analysis of microRNAs was carried out using small RNAs derived from the indicated cell lines. The expression levels of let7-a, U6 and hsa-miR-24 were analyzed as controls. Reaction products were analyzed by 3.0% Nusieve agarose gel electrophoresis. [file 1471-2407-9-198-S12.pdf]

Additional file 13.

**a**

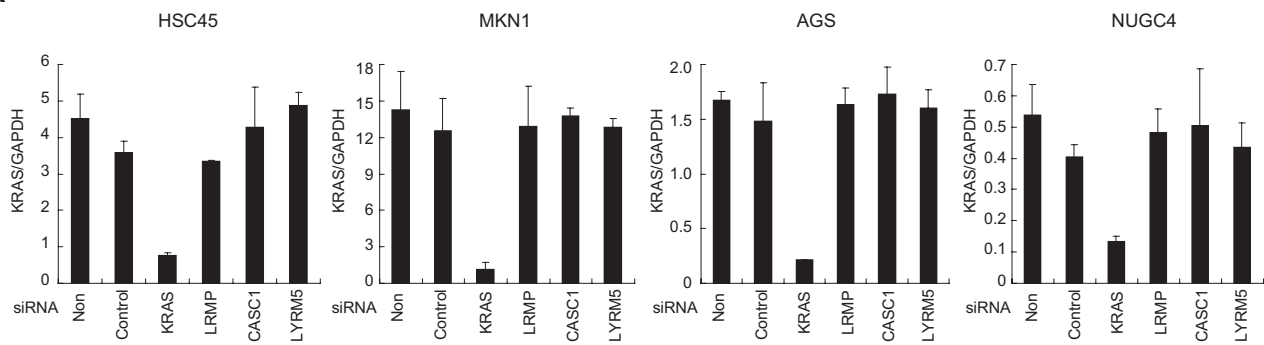

**b**

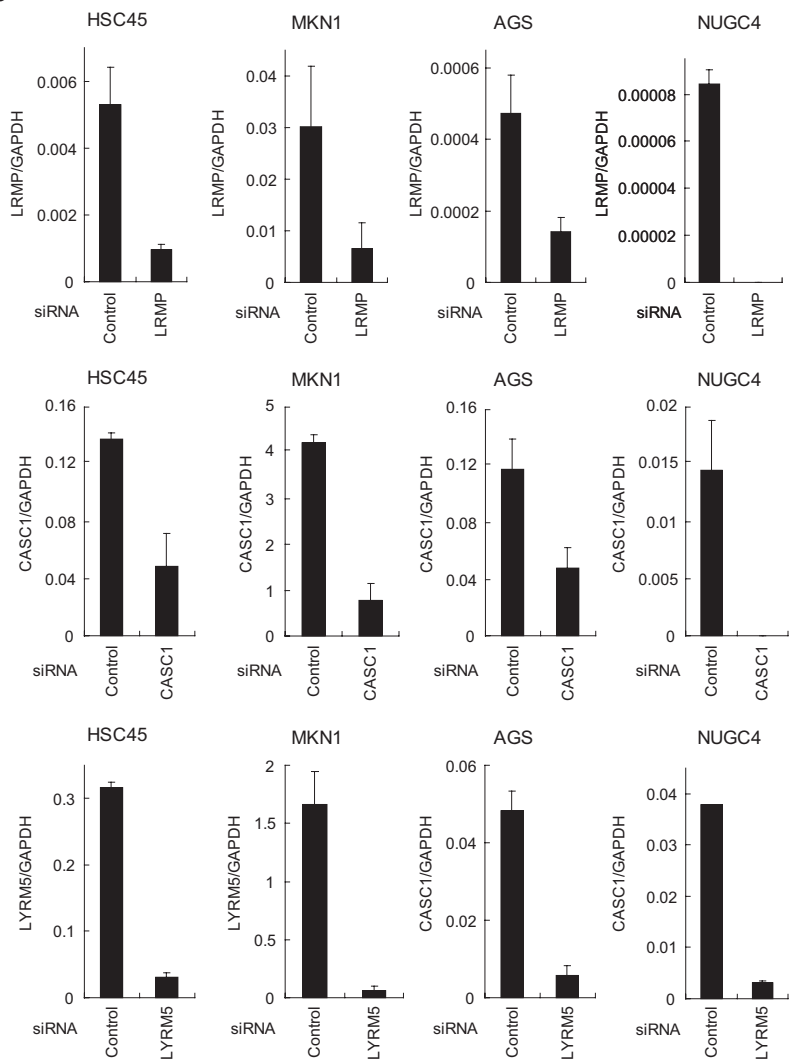

Supplement: Additional file 13 — Gene expression in siRNA knock-down cells. Gastric cancer cell lines were transfected with siRNAs for KRAS, LRMP, CASC1, LYRM5, or a universal non-targeting siRNA as a negative control. Cells were cultured for 48 h and then total RNA was isolated. mRNA expression of KRAS (a) and LRMP, CASC1 and LYRM5 (b) in each cell line was determined by qRT-PCR. The expression of each gene was normalized to that of GAPDH and normal stomach mRNA. Non, nontransfected cells. Data represents the means and SD of three independent experiments. [file 1471-2407-9-198-S13.pdf]
